# *Pseudomonas aeruginosa* inhibits *Rhizopus microsporus* germination through sequestration of free environmental iron

**DOI:** 10.1038/s41598-019-42175-0

**Published:** 2019-04-05

**Authors:** Courtney Kousser, Callum Clark, Sarah Sherrington, Kerstin Voelz, Rebecca A. Hall

**Affiliations:** Institute of Microbiology and Infection, School of Biosciences, University of Birmingham, Birmingham, B15 2TT UK

## Abstract

*Rhizopus spp* are the most common etiological agents of mucormycosis, causing over 90% mortality in disseminated infection. Key to pathogenesis is the ability of fungal spores to swell, germinate, and penetrate surrounding tissues. Antibiotic treatment in at-risk patients increases the probability of the patient developing mucormycosis, suggesting that bacteria have the potential to control the growth of the fungus. However, research into polymicrobial relationships involving *Rhizopus spp* has not been extensively explored. Here we show that co-culturing *Rhizopus microsporus* and *Pseudomonas aeruginosa* results in the inhibition of spore germination. This inhibition was mediated via the secretion of bacterial siderophores, which induced iron stress on the fungus. Addition of *P*. *aeruginosa* siderophores to *R*. *microsporus* spores in the zebrafish larval model of infection resulted in inhibition of fungal germination and reduced host mortality. Therefore, during infection antibacterial treatment may relieve bacterial imposed nutrient restriction resulting in secondary fungal infections.

## Introduction

Mucormycosis is a life threatening, disfiguring infection caused by ubiquitous environmental fungi belonging to the order Mucorales, with *Rhizopus spp*. accounting for approximately 70% of infections^1,2^. In healthy individuals, innate immune cells are capable of controlling spore germination, thus preventing infection^3^. However, patients with uncontrolled diabetes, cancer, neutropenia, burn/traumatic wounds, post-transplantation and those undergoing corticosteroid therapy or renal dialysis are prone to mucormycosis^4,5^. Mucorales are inherently resistant to antifungals, requiring surgical debridement of infected tissue followed by an aggressive antifungal regime. As a result, mucormycosis is associated with high mortality rates (up to 96% in disseminated infections), and significant morbidity^2^.

Mucorales spores enter the body through inhalation or open wounds^6^. As a result mucormycosis is commonly associated with pulmonary, rhinocerebral, or cutaneous infections^7^. Germination is key to the pathogenesis of Mucorales, leading to tissue penetration, endothelial angioinvasion, and vessel thrombosis, ultimately resulting in debilitating necrosis^4^. Traumatic and burn wound infections, including military-associated blast wounds, are known predisposing conditions for mucormycosis in the immunocompetent^2^, with over 70% of these infections being polymicrobial in nature^8,9^. *Pseudomonas aeruginosa*, *Staphylococcus aureus*, and *Escherichia coli* are the most commonly co-isolated bacterial species from chronic wounds^10,11^, and are therefore likely to interact and compete with Mucorales spores. In addition, the emergence of mucormycosis has been associated with broad-spectrum antimicrobial treatment^12–14^, suggesting that the surrounding microbiome plays a role in controlling fungal growth.

Here we show that *P*. *aeruginosa* inhibits the germination, and therefore virulence, of *Rhizopus microsporus*, a common cause of mucormycosis. This inhibition was mainly caused by bacterial secretion of iron-chelating molecules, which sequester iron from the fungus. Considering the prevalence of opportunistic bacteria and Mucorales in traumatic wounds, antibacterial treatment may reduce the presence of nutrient-restricting molecules like bacterial secreted siderophores in the wound environment rendering the environment more permissive to fungal germination, although we acknowledge that other factors including the immune status of the host also play critical roles in controlling fungal infection.

## Results

### *Pseudomonas aeruginosa* inhibits the germination of *Rhizopus microsporus*

**Figure 1:**
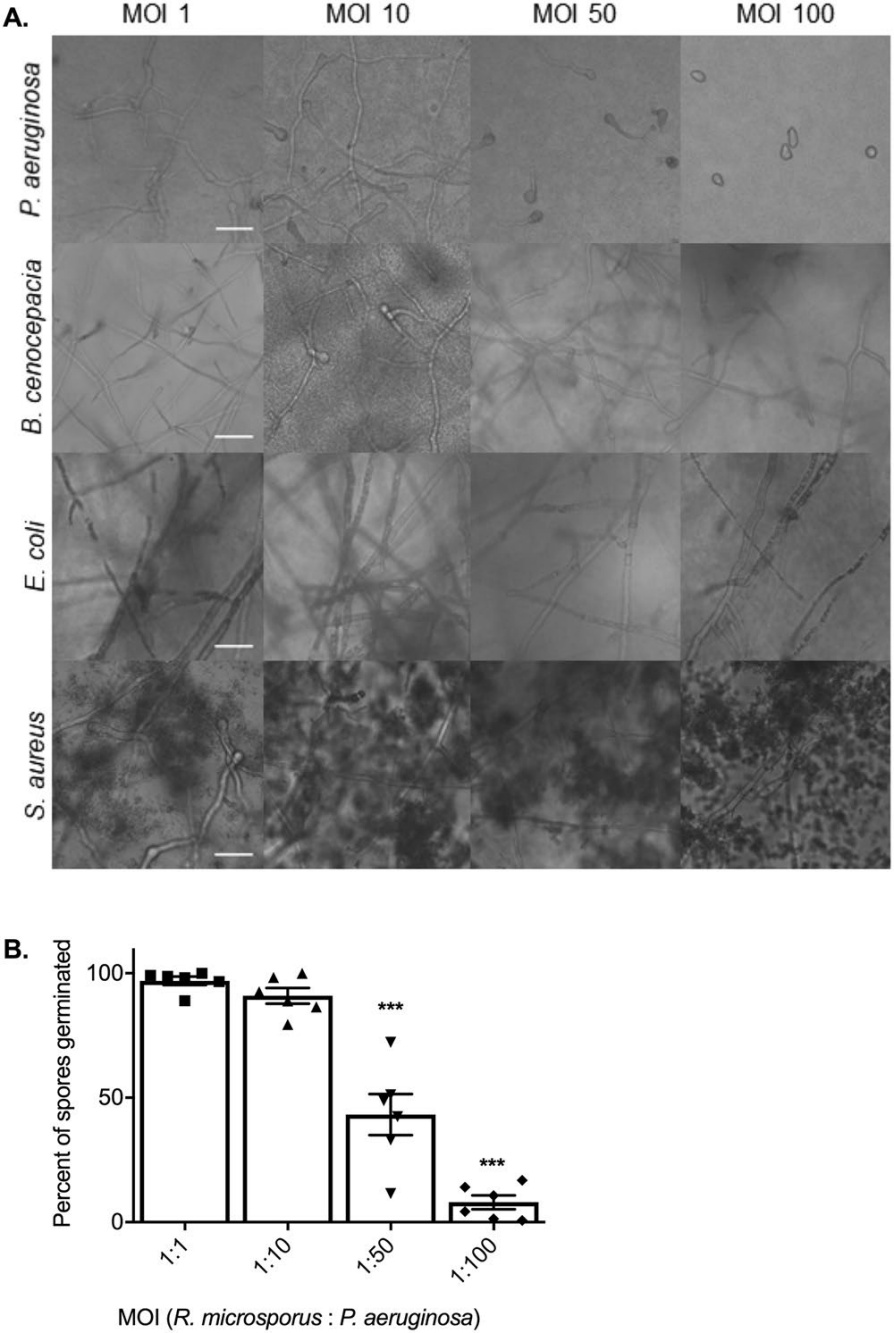
*Pseudomonas aeruginosa* strongly inhibits the germination of *Rhizopus microsporus*. *R*. *microsporus* spores were incubated with live *P*. *aeruginosa*, *B*. *cenocepacia*, *S*. *aureus*, and *E*. *coli* at increasing multiplicities of infection (MOI) for 24 h (**A**) Representative images after 24 h exposure (37 °C, static). Scale bars depict 50 µm. (**B**) Per cent of spores germinated after 24 h exposure to *P*. *aeruginosa*. One-way ANOVA performed on arcsine transformed data (n = 6). ***p < 0.001. Error bars depict SEM.

Key to the pathogenesis of mucormycosis is the ability of spores to germinate and penetrate surrounding tissues^4^. To identify whether bacteria can influence fungal germination, *R*. *microsporus* spores were co-cultured with *Pseudomonas aeruginosa*, *Burkholderia cenocepacia*, *Staphylococcus aureus*, and *Escherichia coli*. Co-culture of *R*. *microsporus* with *P*. *aeruginosa* at multiplicities of infection (MOI) of 1:50 and 1:100 resulted in 56.8% (+/−8.269, p = 0.0023) and 92% (+/−2.784, p < 0.001) inhibition of fungal germination, respectively (Fig. 1A,B). Conversely, co-culturing *R*. *microsporus* spores with *S*. *aureus*, *E*. *coli*, and *B*. *cenocepacia* did not affect fungal growth at any of the MOIs tested (Fig. 1A). Taken together, the results obtained from live co-cultures between *R*. *microsporus* and *P*. *aeruginosa* indicate that these two microbes undergo a competitive relationship resulting in reduced fungal germination.

### *P*. *aeruginosa* inhibits spore germination through secreted factors

**Figure 2:**
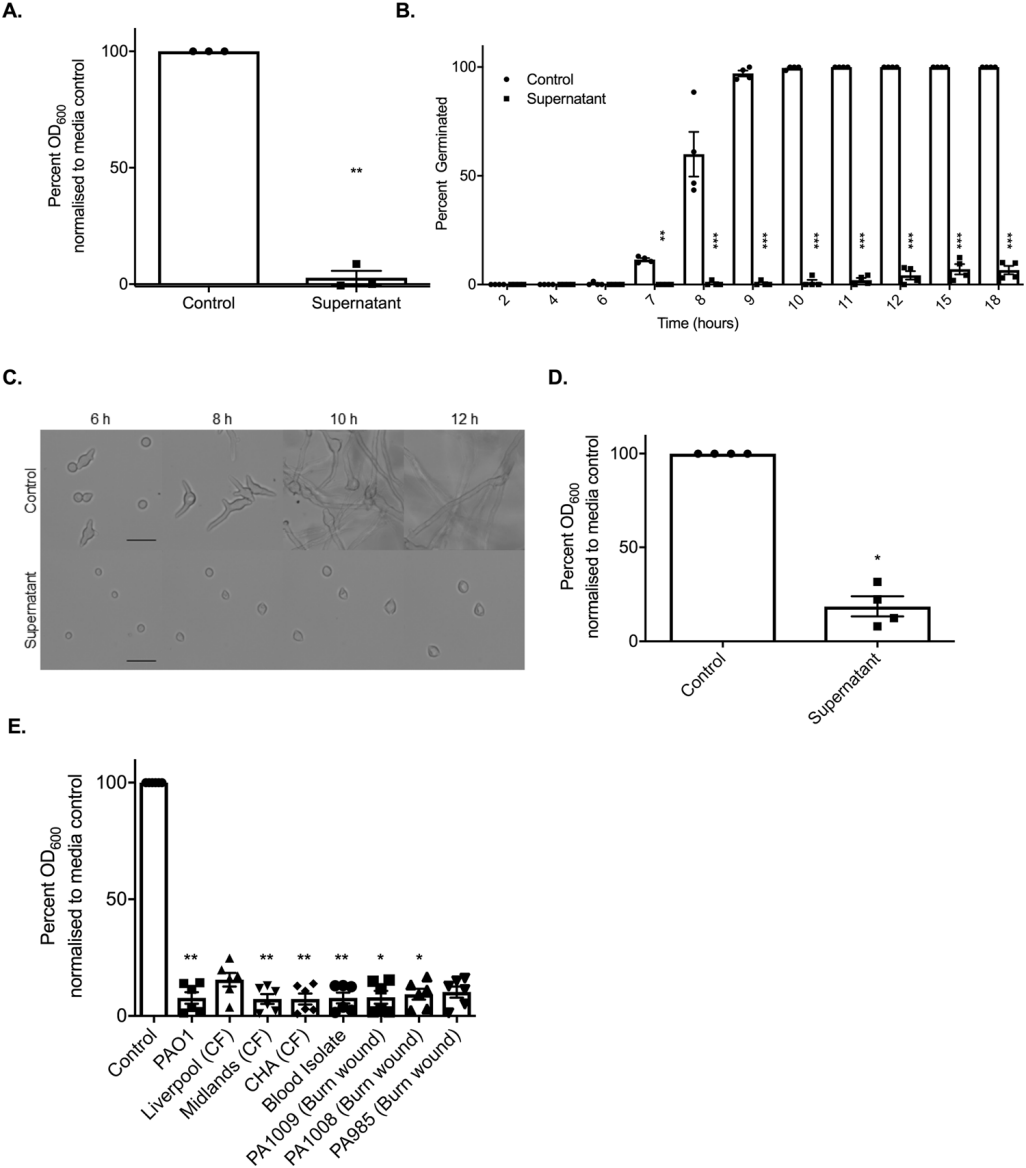
*P*. *aeruginosa* inhibits spore germination through secreted factors. *R*. *microsporus* spores were exposed to 50% *P*. *aeruginosa* PAO1 supernatant for 24 h. (**A**) Fungal growth was measured through absorbance (OD_600_) and normalised to media control (n = 3). To determine the point of inhibition, spore germination was observed via live-cell imaging and (**B**) the per cent of spores germinated over time was quantified (n = 4, Two-way ANOVA performed on arcsine transformed data). (**C**) Representative images of spores germinating over time were collected. Scale bar = 50 µm. (**D**) To determine whether the supernatant also inhibits the continuation of growth after germination is initiated, spores were incubated in SAB for 4–5 h until germlings emerged, and subsequently exposed to 50% PAO1 supernatant for 18 h (n = 4, Mann-Whitney *U* test). (**E**) To test whether this is a lab strain-specific phenomenon, *R*. *microsporus* spores were exposed to supernatants from *P*. *aeruginosa* clinical isolates for 24 h (n = 6). Fungal growth was determined through absorbance (OD_600_). All data was analysed by a Kruskal-Wallis test with Dunn’s multiple comparisons test unless indicated otherwise. *p < 0.05, **p < 0.01, ***p < 0.001. Error bars depict SEM.

Microbes are able to communicate through the secretion of secondary metabolites, quorum sensing molecules, and metabolic by-products^15–20^. Therefore, to deduce whether the observed inhibition of *R*. *microsporus* germination was a result of direct cell-cell interactions or mediated through secreted products, *R*. *microsporus* spores were incubated in *P*. *aeruginosa* spent culture supernatants. Incubation of *R*. *microsporus* spores with 50% *P*. *aeruginosa* supernatant resulted in 94.4% (+/−0.01769, p = 0.0022) inhibition of fungal growth (Fig. 2A), confirming that the inhibitory molecule(s) are secreted by *P*. *aeruginosa*. Time-lapse microscopy confirmed that the presence of the supernatant resulted in a significant reduction in spore germination (Fig. 2B,C, Videos 1 and 2), with only 6.7% (+/−3.8, p < 0.0001) of spores germinating after 18 h. However, the inhibition of germination did not affect spore swelling (Videos 1 and 2). To deduce whether *P*. *aeruginosa* supernatants are able to inhibit fungal growth after the initiation of germination, spores were pre-germinated, and then subsequently incubated with 50% supernatant. Fungal growth was significantly reduced (by 81.4% +/− 5.252, p = 0.0286) in the presence of the supernatant compared to the media control (Fig. 2D). Therefore, *P*. *aeruginosa* supernatants are able to inhibit both germination and growth of *R*. *microsporus*.

To determine whether the inhibitory molecule(s) is produced by other *P*. *aeruginosa* strains, we tested the ability of supernatants from a series of *P*. *aeruginosa* isolates to inhibit spore germination. *R*. *microsporus* germination was inhibited in the presence of supernatants from all *P*. *aeruginosa* clinical isolates (Fig. 2E), suggesting that the production of this inhibitory molecule is a general trait of *P*. *aeruginosa* and is not limited to laboratory-evolved strains.

As fungal germination is dependent on environmental pH and nutrient availability^21,22^, we assessed whether the addition of the supernatant was inhibiting germination through modulation of these parameters. Addition of the bacterial supernatant to SAB broth resulted in mild alkalisation of the media (pH 7.33 vs. 6.45). However, adjusting the pH of the control media to pH 7.33, to mimic the conditions in media containing the *P*. *aeruginosa* supernatant, did not affect *R*. *microsporus* germination rates (Supplementary Fig. S1). To elucidate the role of macronutrient restriction, SAB broth was diluted with 50% phosphate buffered solution (PBS) to mimic the nutrient limitation imposed by the addition of 50% supernatant. However, the spores were still able to germinate under these conditions (Supplementary Fig. S1). Therefore, *P*. *aeruginosa* secretes a molecule(s) that is able to inhibit *R*. *microsporus* germination independent of pH and macronutrient limitation.

### Inhibition of *R*. *microsporus* germination is not mediated by quorum sensing molecules or pyocyanin

Bacteria secrete a diverse range of proteins and secondary metabolites to aid in host colonisation and inter-species competition. To determine whether the secreted factor responsible for inhibiting spore germination is proteinaceous, *P*. *aeruginosa* supernatants were boiled or treated with Proteinase K to degrade any secreted proteins. Supernatants that were boiled or treated with Proteinase K inhibited *R*. *microsporus* growth (97.62%, +/−1.558, p = 0.0355 and 99.03%, +/−1.634, p = 0.0140, respectively) (Supplementary Fig. S2A), suggesting that a secreted, heat-stable molecule mediates the observed inhibition of *R*. *microsporus* germination.

*P*. *aeruginosa* secretes several heat-stable cell density-dependent signalling molecules into the environment to regulate virulence by sensing population density and inducing the expression or inhibition of population-dependent mechanisms^23^. These quorum sensing molecules (QSMs) are well known to regulate intra- and inter-species interactions including inhibiting the morphological switch of *Candida albicans*^24–27^. Therefore, we tested the ability of the major *P*. *aeruginosa* QSMs to inhibit *R*. *microsporus* germination. Exposure of *R*. *microsporus* spores to N-butanoyl-l-homoserine lactone (C4 HSL), N-hexanoyl-DL-homoserine lactone (C6 HSL), and N-octanoyl-L-homoserine lactone (C8 HSL), did not affect fungal growth (Supplementary Fig. S2B–D). At high concentrations (200 μM) N-(3-oxododecanoyl)-L-homoserine lactone (C12 HSL) resulted in 42.1% (+/−0.1518, p = 0.1331) reduction in fungal growth (Supplementary Fig. S2E). Therefore, secreted QSMs appear to not be the major regulators of *R*. *microsporus* growth.

Pyocyanin is a heat stable, secreted blue-pigmented toxin, which is known to increase the virulence of *P*. *aeruginosa* by depressing the host immune responses through induction of neutrophil apoptosis^28,29^. Pyocyanin also inhibits the growth and morphogenesis of *C*. *albicans* and *Aspergillus fumigatus*^30^. Therefore, we determined whether the presence of pyocyanin in the supernatant was inhibiting the germination of *R*. *microsporus*. Addition of purified pyocyanin resulted in 31.4% (+/−0.1434, p > 0.9999) inhibition of *R*. *microsporus* growth at concentrations above 100 μM (Supplementary Fig. S2F). To deduce whether these pyocyanin concentrations were physiologically relevant the concentration of pyocyanin in the *P*. *aeruginosa* supernatants was quantified through absorbance measurement (690 nm) and compared to a standard curve of pre-defined pyocyanin concentrations. Growth of *P*. *aeruginosa* in LB media at 200 rpm, 37 °C did not result in the secretion of detectable levels of pyocyanin (not shown). Therefore, the inhibition of *R*. *microsporus* growth in the *P*. *aeruginosa* supernatant was not due to pyocyanin.

To determine whether the secreted factor is a lipophilic molecule, chloroform extractions were performed. The organic phase of the supernatant did not significantly inhibit spore germination (18% inhibition, +/−3.058, p = 0.0926), while the aqueous phase maintained its inhibitory action (95.1% inhibition, +/−0.9559, p = 0.0079, Supplementary Fig. S2G). Therefore, a secreted, heat-stable, water-soluble molecule(s) inhibits the growth of *R*. *microsporus*.

### *P*. *aeruginosa* inhibits *R*. *microsporus* germination via iron sequestration

**Figure 3:**
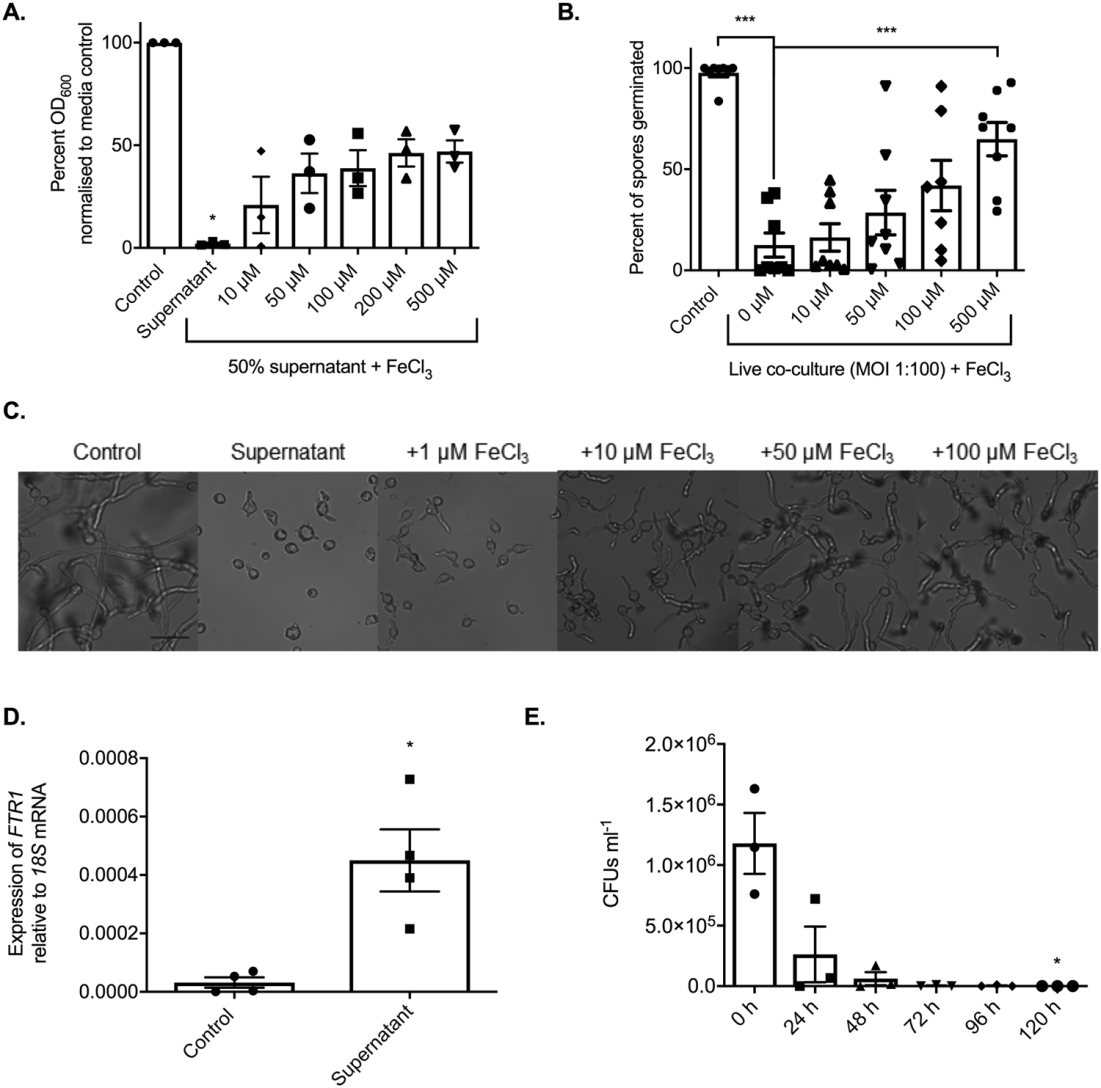
*P*. *aeruginosa* inhibits *R*. *microsporus* germination via iron sequestration. *R*. *microsporus* spores were exposed to (**A**) 50% *P*. *aeruginosa* supernatant and spiked with increasing concentrations of iron chloride for 24 h statically at 37 °C. Fungal growth was measured through absorbance (OD_600_) and normalised to media control (n = 3, Kruskal-Wallis test with Dunn’s multiple comparisons test). (**B**) This ability to rescue was confirmed in a live co-culture setting, where the addition of exogenous iron increased the per cent of spores germinated after 24 h in a dose-dependent manner (n = 8). As the addition of iron in 50% supernatant increased overall growth, (**C**) representative images were collected at 9 h to confirm ability to rescue germination. Scale bar depicts 50 µm. (**D**) Iron starvation of *R*. *microsporus* spores after 7 h exposure to *P*. *aeruginosa* supernatant was determined through strong upregulation of the high-affinity iron permease *FTR1* (n = 4, Mann-Whitney *U* test). (**E**) As iron starvation is associated with Mucorales apoptosis, the viability of spores exposed to 100% *P*. *aeruginosa* supernatant over time was quantified by counting colony forming units (CFUs) every 24 h for 120 h (n = 3, Kruskal-Wallis test with Dunn’s multiple comparisons test). *p < 0.05, ***p < 0.001.

Research has established the importance of metal micronutrients for microbial growth and pathogenicity, and the ability of host metal sequestering proteins to inhibit both fungal and bacterial growth through nutritional immunity^31–33^. Iron, zinc, copper, and manganese are considered the most important trace metals for the growth of fungi and the availability of iron is key to the pathogenesis of Mucorales^34,35^. Therefore, we investigated whether the supernatants were imposing micronutrient restriction on *R*. *microsporus*. Supplementing the supernatants with iron was able to partially restore fungal growth, resulting in 46.2% *R*. *microsporus* growth at concentrations above 200 µM (+/−6.660, Fig. 3A,C) and an insignificant difference as compared to the control (p > 0.9999). However, supplementation with zinc, copper or manganese did not rescue *R*. *microsporus* growth (Supplementary Fig. S3). Therefore, the majority of growth inhibition appears to result from the *P*. *aeruginosa* supernatants specifically sequestering iron from the environment. However, we acknowledge that other unidentified factors may play a role as supplementation with iron did not completely restore fungal growth. To confirm that the inhibition of spore germination observed in the co-cultures was also attributed to iron restriction, co-cultures of *R*. *microsporus* and *P*. *aeruginosa* were spiked with iron. Germination and therefore growth of *R*. *microsporus* in the co-culture was recovered at concentrations above 100 µM (41.9% +/− 12.46, p = 0.1540) to levels comparable to those observed for iron spiked supernatants (Fig. 3B), confirming that in both scenarios the major contributing factor, resulting in the inhibition of fungal growth, appears to be the sequestration of iron by *P*. *aeruginosa*.

Iron starvation has previously been shown to up-regulate the high affinity iron permease *FTR1* in other *Rhizopus* species^36^. Therefore, to confirm that *R*. *microsporus* is undergoing iron starvation in the presence of *P*. *aeruginosa* supernatants, the expression levels of *FTR1* were determined by qRT-PCR. *FTR1* was highly upregulated (10-fold increase, p = 0.0286) when exposed to 50% *P*. *aeruginosa* supernatant for 7 h, as compared to the control (Fig. 3D). This confirms that *P*. *aeruginosa* mediated iron restriction inhibits *R*. *microsporus* growth and germination.

Iron starvation has been shown to induce apoptosis in *R*. *oryzae* after prolonged starvation^36^. Therefore, if spores are undergoing iron starvation when exposed to *P*. *aeruginosa* supernatant, prolonged exposure should decrease survival. To isolate the effects of the supernatant, we used 100% *P*. *aeruginosa* supernatant to monitor spore survival over time. In this condition, the viability of spores was reduced by 82.40% (+/−13.44) after 24 h, and no viable spores were recovered after 120 h (p = 0.0490, Fig. 3E). This indicates that iron is essential for the survival and pathogenicity of *R*. *microsporus*.

**Figure 4:**
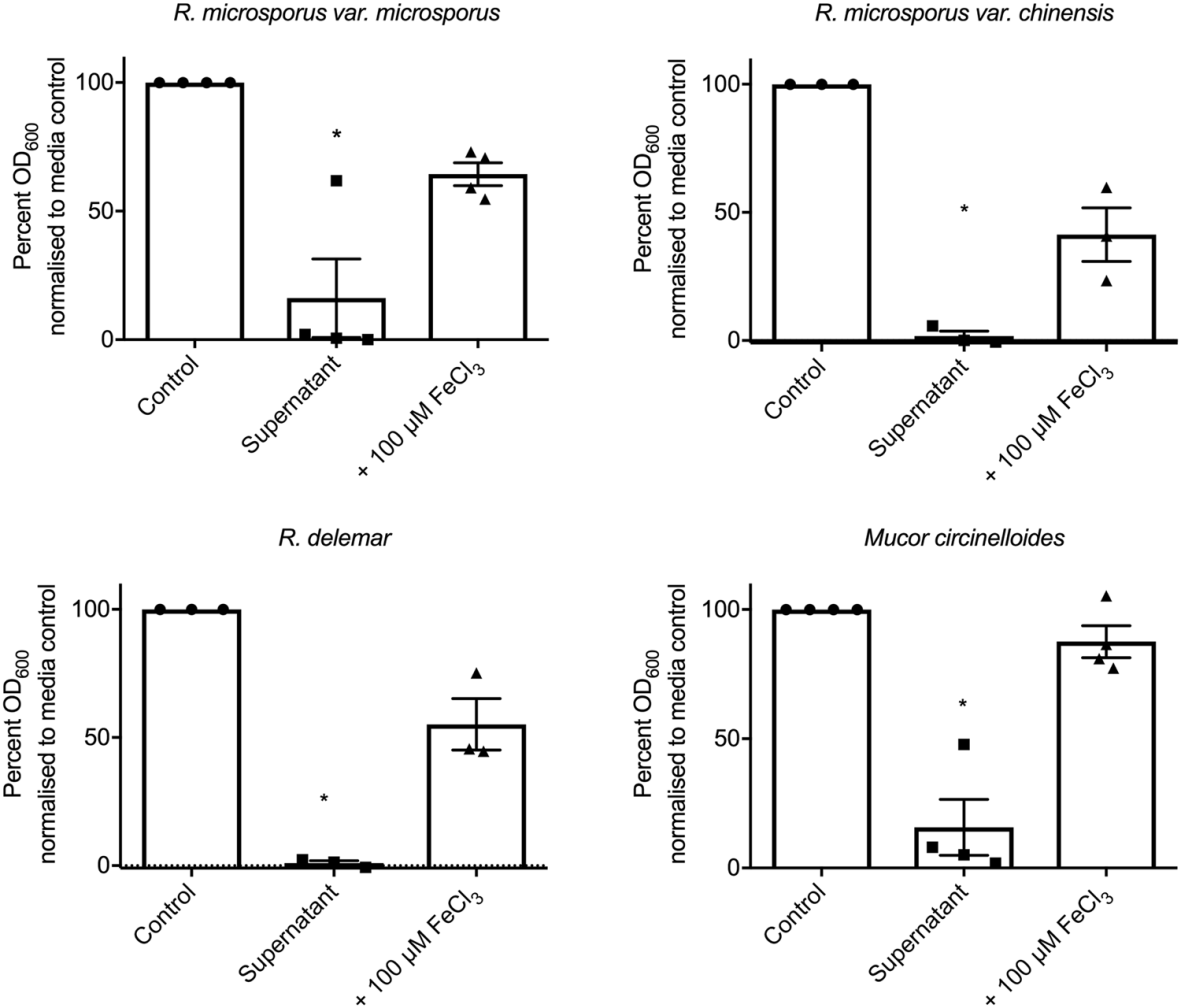
Iron-dependent inhibition of Mucorales by *P*. *aeruginosa* is not *R*. *microsporus* strain-specific. Most experiments in this study were performed using an *R*. *microsporus* clinical isolate. To ensure the inhibitory effect of *P*. *aeruginosa* is not limited to this isolate, *R*. *microsporus var*. *microsporus*, *R*. *microsporus var*. *chinensis*, *R*. *delemar*, and *Mucor circinelloides* were exposed to 50% *P*. *aeruginosa* supernatant with and without the addition of 100 µM FeCl_3_. Fungal growth was determined at 24 h by measuring absorbance (OD_600_) and normalising to control (n = 3). *p < 0.05. All data was analysed by a Kruskal-Wallis test with Dunn’s multiple comparisons test.

To delineate whether bacteria-associated iron restriction inhibits the growth of Mucorales in general, we tested the ability of *P*. *aeruginosa* supernatant to inhibit the growth of *R*. *microsporus var*. *microsporus*, *R*. *microsporus var*. *chinensis*, *R*. *delemar*, *and Mucor circinelloides*. The growth of all isolates was significantly reduced in the presence of *P*. *aeruginosa* supernatant [83.85% (+/−15.23), 98.26% (+/−1.977), 99.01% (+/−0.8695), and 87.54% (+/−10.78), respectively], and was significantly rescued by the addition of 100 μM of iron [64.36% (+/−4.450), 41.30% (+/−10.48), 55.14% (+/−10.01), and 87.54% (+/−6.219), respectively]. This confirms that the bacteria-associated inhibition of growth is a general trait of Mucorales and highlights the potential differences between Mucorales strains in response to bacteria (Fig. 4).

### Siderophore deficient *P*. *aeruginosa* strains lack the ability to suppress germination

**Figure 5:**
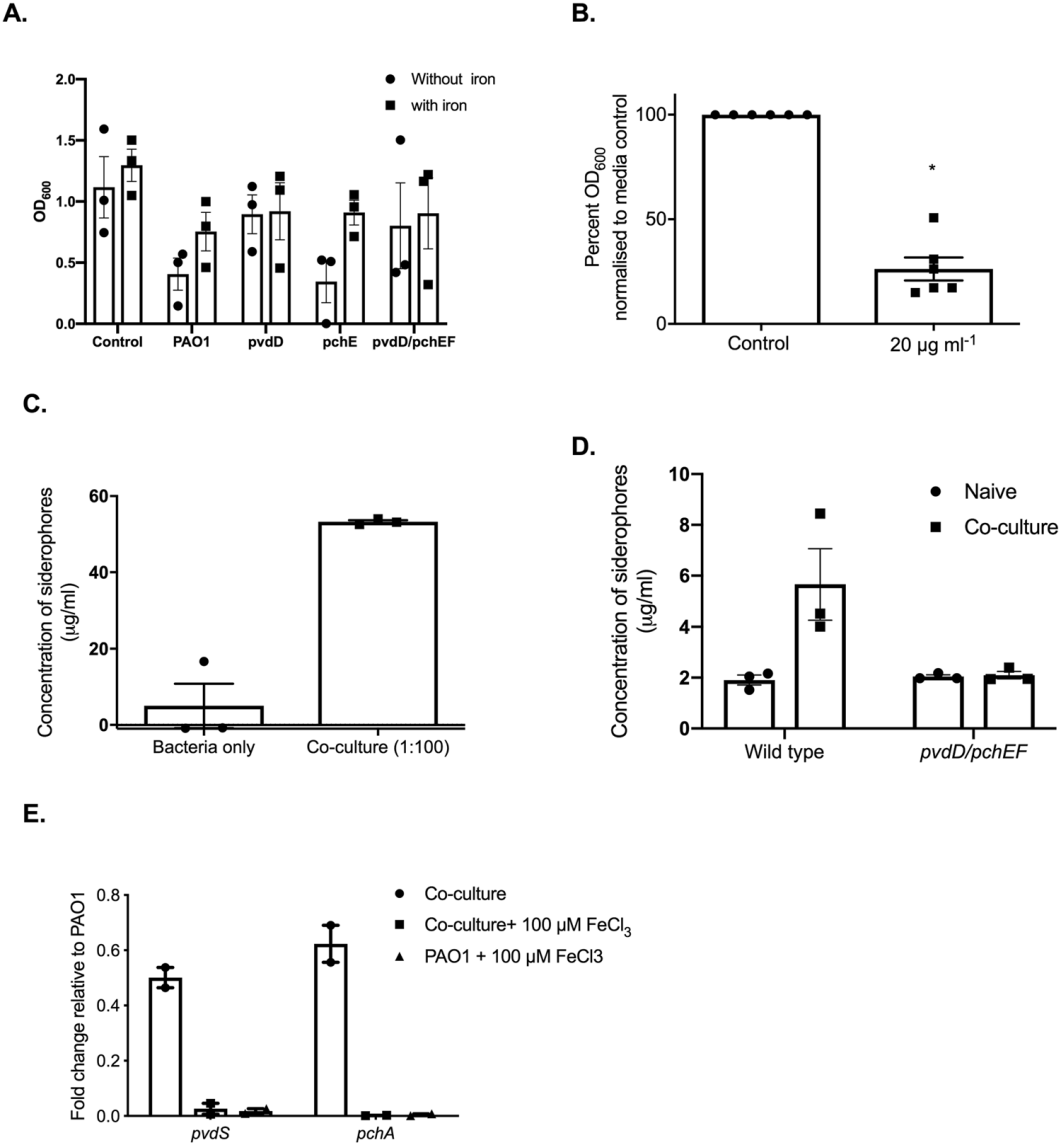
*P*. *aeruginosa*-imposed iron restriction is largely mediated via pyoverdine production. (**A**) *R*. *microsporus* was grown in bacterial supernatants from wild type *P*. *aeruginosa*, strains defective in siderophore biosynthesis, or standard LB mixed 25:75 with SAB, with and without iron. *R*. *microsporus* spores were exposed to these mixtures for 24 h and fungal growth was determined via absorbance (OD_600_, n = 3). (**B**) *R*. *microsporus* spores were incubated in SAB with 20 μg ml^−1^ of exogenous pyoverdine at 37 °C for 24 h. Fungal growth was measured through absorbance (OD_600_) and normalised to media control (n = 6). (**C**) *P*. *aeruginosa* was exposed to *R*. *microsporus* at an MOI of 1:100 (*R*. *microsporus:P*. *aeruginosa*) for 24 h (37 °C). Siderophore production was measured by using the Siderotec Assay (EmerginBio) (n = 3, Mann-Whitney *U* test). (**D**) *P*. *aeruginosa* strains defective in siderophore biosynthesis were exposed to *R*. *microsporus* at an MOI of 1:100 (*R*. *microsporus:P*. *aeruginosa*) for 24 h (37 °C). Siderophore production was measured by using the Siderotec Assay (EmerginBio) (n = 3, Mann-Whitney *U* test). (**E**) *P*. *aeruginosa* was exposed to *R*. *microsporus* at an MOI of 1:100 (*R*. *microsporus:P*. *aeruginosa*) for 7 h, snap frozen and total RNA extracted. The expression levels of *PvdS* and *PchA* were quantified by qRT-PCR relative to *RpoD* and normalised to PAO1 grown in isolation.

Iron sequestering is mediated via iron binding proteins and molecules known as siderophores. Pyoverdine and pyochelin are the two predominate siderophores produced by *P*. *aeruginosa*, with pyoverdine exhibiting the highest affinity for iron^37,38^. To identify the role of these siderophores in this interaction, we quantified fungal growth in the presence of the supernatants from *P*. *aeruginosa* strains deficient in either siderophore alone, or in combination. Growth of *R*. *microsporus* was inhibited when incubated with culture supernatants from *P*. *aeruginosa* strains defective in pyochelin biosynthesis (∆*pchEF*), with growth being rescued by exogenous iron (Fig. 5A), suggesting that this siderophore plays a minor role in sequestering iron in these experiments. However, *R*. *microsporus* germinated in the presence of bacterial supernatants from *P*. *aeruginosa* mutants defective in pyoverdine biosynthesis (∆*pvdD*) or in pyoverdine and pyochelin biosynthesis (∆*pchEF∆pvdD*) (Fig. 5A), confirming that, under the tested conditions, *P*. *aeruginosa* imposed iron restriction is largely mediated by the secretion of pyoverdine.

To confirm that pyoverdine alone is sufficient to inhibit *R*. *microsporus* growth, spores were exposed to exogenous pyoverdine. *P*. *aeruginosa* supernatants contained 58.9 µg ml^−1^ (+/−1.194) siderophores, making the concentration of siderophores in our assay 29.45 µg ml^−1^. Therefore, *R*. *microsporus* spores were grown in the presence of 20 µg ml^−1^ purified pyoverdine to resemble siderophore concentrations similar to the culture supernatants. Incubation of fungal spores with pyoverdine significantly reduced fungal growth (73.8%, +/−5.503, p = 0.0022, Fig. 5B). Therefore, pyoverdine alone is sufficient to inhibit *R*. *microsporus* growth and germination.

### *R*. *microsporus* induces iron stress and promotes bacterial siderophore production

*C*. *albicans* can decrease *P*. *aeruginosa* siderophore production through suppression of the pyoverdine and pyochelin biosynthetic pathways^19^. To determine whether *R*. *microsporus* is also able to interfere with *P*. *aeruginosa* siderophore production, the concentration of siderophores after 24 h mono- and co-culture was quantified (Fig. 5C). Surprisingly, the concentration of siderophores in mono-cultures in SAB/LB was lower than in LB (4.99 μg ml^−1^, +/−5.834, vs 58.9 µg ml^−1^ (+/−1.194)) suggesting that LB/SAB has a higher iron content, reducing siderophore production. However, in co-cultures, siderophore levels were increased to levels similar to the LB supernatant (53.25 μg ml^−1^, +/−0.4335 compared to 58.9 µg ml^−1^ (+/−1.194) indicative of imposed iron stress. This increase in siderophore concentration was not observed in co-cultures containing siderophore deficient *P*. *aeruginosa* (Fig. 5D), confirming that the increase in siderophore concentration is likely due to increased bacterial rather than fungal siderophore biosynthesis. In agreement with this, key genes involved in pyoverdine and pyochelin biosynthesis were upregulated during co-culture with *R*. *microsporus*. However, this regulation was lost in the presence of exogenous iron (Fig. 5E). These results confirm that during co-culture, the two organisms compete for iron, resulting in the upregulation of bacterial siderophore biosynthesis and *P*. *aeruginosa* outcompeting *R*. *microsporus* for iron and therefore growth.

### The concentration of siderophores produced by bacteria correlates with inhibition of *R*. *microsporus* growth

**Figure 6:**
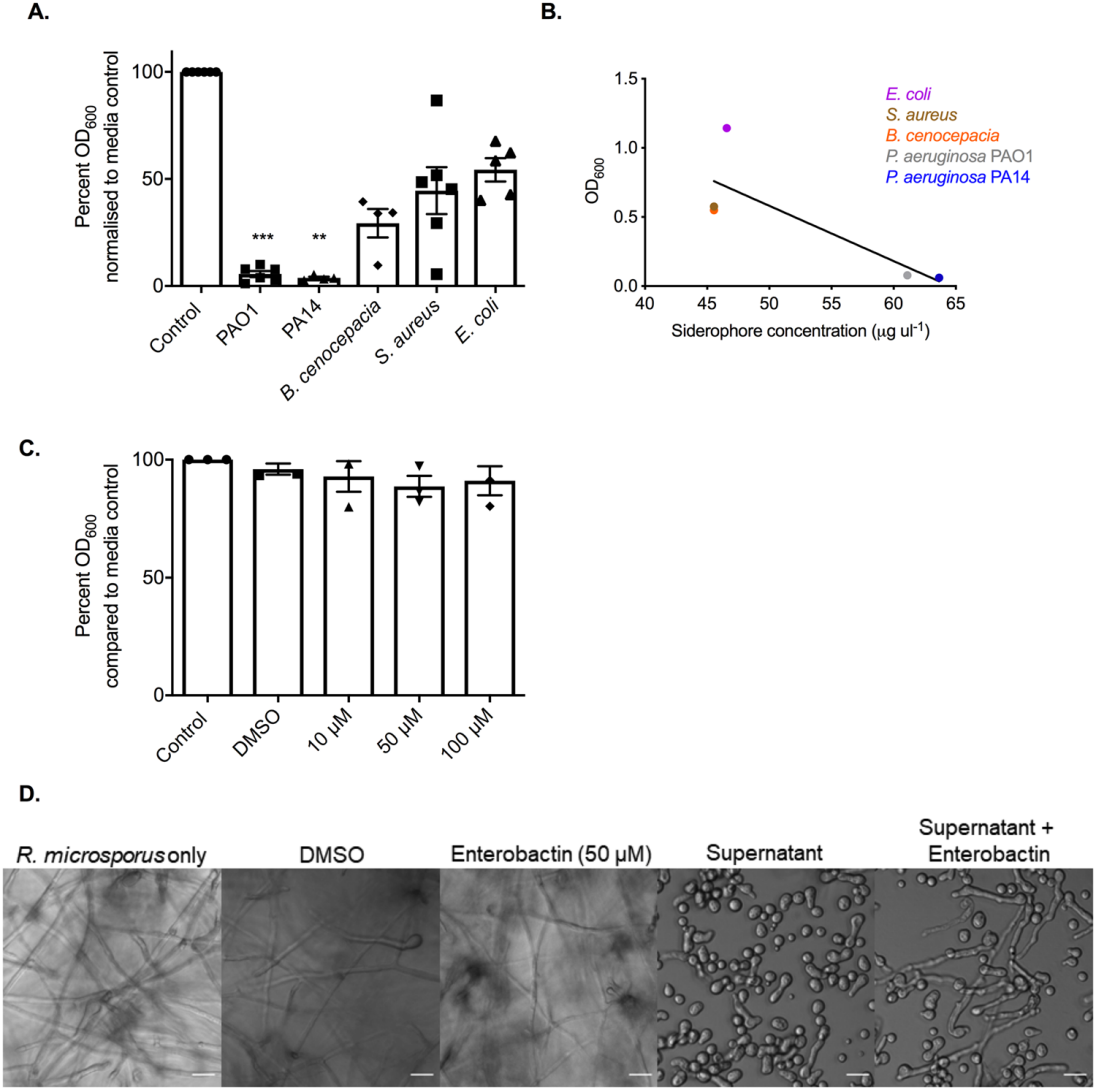
*R*. *microsporus* can utilise enterobactin as a xenosiderophore. (**A**) *R*. *microsporus* spores were exposed to 50% supernatants harvested from *P*. *aeruginosa* PAO1, *P*. *aeruginosa* PA14, *B*. *cenocepacia*, *S*. *aureus*, and *E*. *coli* for 24 h. Fungal growth was determined by absorbance (OD_600_) and normalised to control. (n = 6). (**B**) Concentration of siderophores produced by bacteria was determined by using the Siderotec Assay (EmerginBio). Correlation between total siderophore production and fungal growth was determined by performing a linear regression with Pearson correlation (n = 3). (**C**) *R*. *microsporus* spores were exposed to varying concentrations of purified enterobactin for 24 h at 37 °C (n = 3). Fungal growth was determined by absorbance (OD_600_) and normalised to control. (**D**) Enterobactin was added to PAO1 sterile supernatants diluted 50% with SAB to a final concentration of 50 μM and incubated at 37 °C for 24 h. Scale bar represents 20 μm. All data was analysed by a Kruskal-Wallis test with Dunn’s multiple comparisons test unless indicated otherwise. *p < 0.05, **p < 0.01, ***p < 0.001.

While live *E*. *coli*, *B*. *cenocepacia*, and *S*. *aureus* did not inhibit the growth of *R*. *microsporus*, these bacteria all produce siderophores^39–41^. To determine the ability of secreted factors to inhibit growth, *R*. *microsporus* spores were exposed to sterile supernatants from *E*. *coli*, *B*. *cenocepacia* and *S*. *aureus* to determine their ability to inhibit *R*. *microsporus* growth as compared to *P*. *aeruginosa* PAO1 and PA14. Consistent with the co-culture experiments, *P*. *aeruginosa* was the only supernatant able to significantly inhibit growth (Fig. 6A). We further investigated whether this lack of inhibition was associated with insufficient production of iron binding molecules by measuring the amount of siderophores produced after 24 h growth in LB. There was a negative correlation between fungal growth and siderophore production across different bacterial species (p = 0.0029, Fig. 6B), suggesting that siderophore mediated iron restriction may be a common mechanism of bacteria to compete with fungi. However, the presence of supernatants from *E*. *coli* had no effect on fungal growth. This was surprising, as *E*. *coli* produces enterobactin, a siderophore with a high affinity (10^52^ M) for iron^42^. In agreement with this data, exogenous enterobactin did not inhibit *R*. *microsporus* growth (91%, +/−6.170, p = 0.936, Fig. 6C), suggesting that *R*. *microsporus* may utilise enterobactin as a xenosiderophore. Therefore, the addition of enterobactin in the presence of *P*. *aeruginosa* may provide an advantage to *R*. *microsporus*. To explore this possibility, we added exogenous enterobactin to *R*. *microsporus*-*P*. *aeruginosa* co-cultures. However, the presence of the enterobactin appeared to enhance the growth of the *P*. *aeruginosa*, presumably because *P*. *aeruginosa* can also utilise enterobactin as a xenosiderophore. Therefore, instead we added enterobactin to sterile bacterial supernatants. *R*. *microsporus* displayed increased germination in *P*. *aeruginosa* supernatants supplemented with enterobactin, although fungal growth was not fully restored (Fig. 6D), presumably due to pyoverdine and pyochelin binding the majority of the free iron. Therefore, taken together this data indicates that *R*. *microsporus* can use enterobactin as a xenosiderophore.

### The bacterial siderophore, pyoverdine, reduces the virulence of *R*. *microspores*

**Figure 7:**
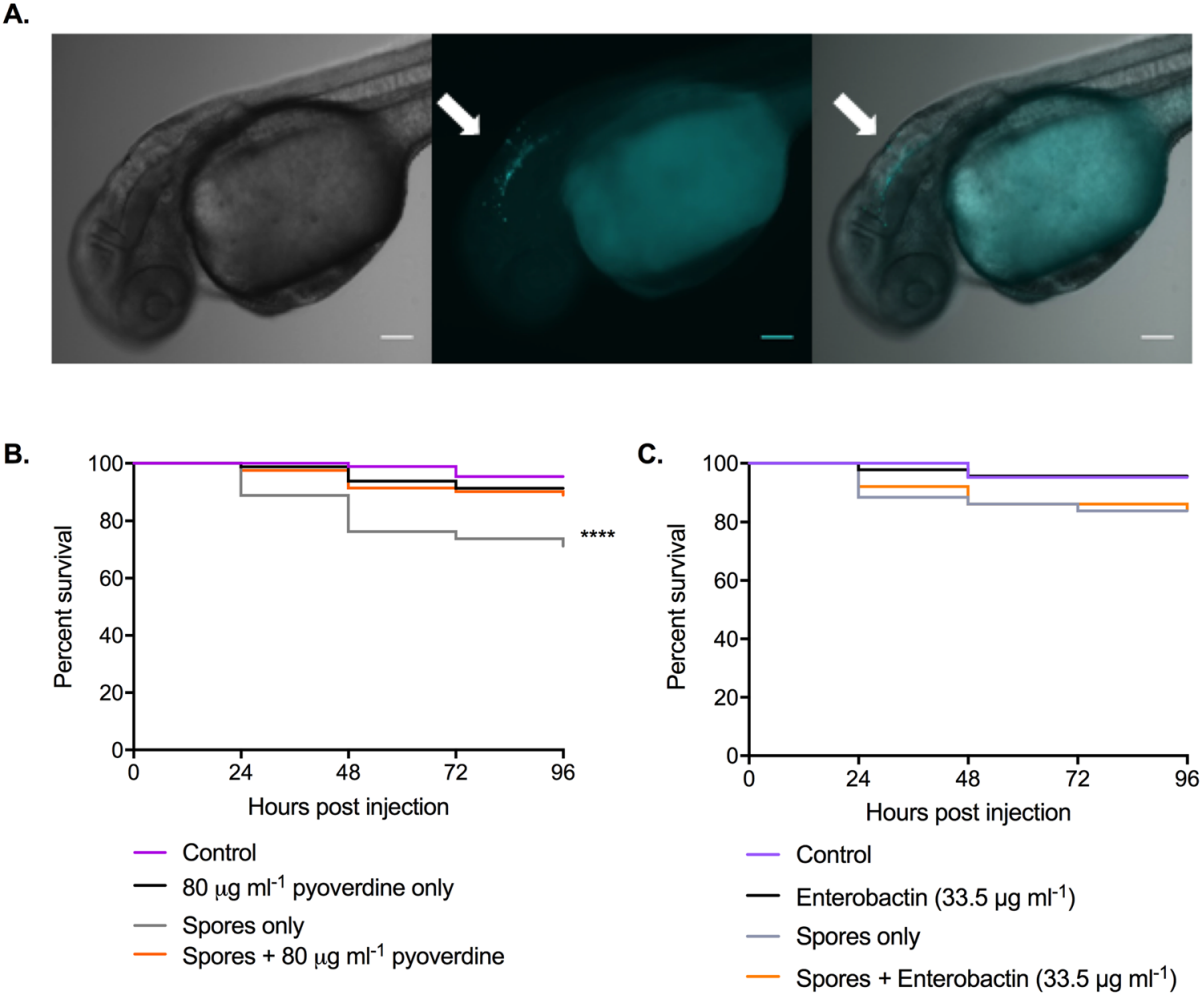
The bacterial siderophore, pyoverdine, reduces *R*. *microsporus* virulence in a zebrafish model of infection. To determine the impact of pyoverdine on fungal virulence within a host, zebrafish larvae were injected in the hindbrain with 50 spores +/−80 μg ml^−1^ pyoverdine. (**A**) Representative images of zebrafish larvae at 0 hpi. White arrows indicate *R*. *microsporus* spores (Calcofluor White stain, cyan pseudo-coloured) located within hindbrain compartment. Scale bars depict 100 μm. (**B**) Survival of larvae was quantified over time. Shown are data pooled from four separate experiments with a total of 87, 80, 80, and 81 fish for control, pyoverdine only, spores only, and spores + pyoverdine, respectively. Data analysed with Mantel-Cox log-rank test. (**C**) Survival of larvae was quantified over time. Data shown is pooled from two independent experiments with a total of 42, 44, 46, 43, and 50 fish for control, enterobactin only, spores only, and spores + enterobactin, respectively. Data analysed with Mantel-Cox log-rank test.

To determine whether the effects of the bacterial siderophores have a role in controlling fungal infection in the host, we utilised the zebrafish larval model (Fig. 7A). Co-injection of *R*. *microsporus* with pyoverdine (80 µg ml^−1^) resulted in a mild but significant increase in fish survival when compared to spores alone (Fig. 7B) with 89% (+/−6.377) of fish surviving across a 96-h time course. As a control to rule out any impact of the siderophore on innate immune cell function, fish were also infected with *R*. *microsporus* in the presence of enterobactin, which should induce iron restriction on the host, but not on the pathogen due to its ability to use enterobactin as a xenosiderophore. Unlike pyoverdine, the addition of enterobactin did not increase the survival of the larvae compared to larvae infected with *R*. *microsporus* alone (Fig. 7C). Together these data confirm that the presence of pyoverdine is sufficient to reduce host damage caused by *R*. *microsporus* infection.

## Discussion

Mucormycosis is a lethal infection with high mortality rates and lack of treatment options due to intrinsic antifungal resistance^43^. Our current understanding of the pathogenesis is incomplete, especially when compared to other opportunistic fungal pathogens such as *C*. *albicans* and *A*. *fumigatus*. Because of this, it is important to understand the pressures Mucorales encounter within the human body. This not only includes pressures from the host, but also from the microbiota. Here we identify that *P*. *aeruginosa* is able to inhibit the germination of *R*. *microsporus* through the secretion of siderophores.

*Pseudomonas* species interact with and control the growth of a variety of fungal species including important plant and animal pathogens^26,44,45^. These interactions have been linked to a variety of contact dependent^46^ and bacterial secreted factors^26,44,45^. The most characterised secreted molecules known to affect fungal growth and morphology are the phenazines^47,48^ and the homoserine lactones^26,44^. For example, in *C*. *albicans* low levels of phenazines inhibit filamentation and biofilm formation, and are fungicidal at high concentrations^49^. Furthermore, the quorum sensing molecule, 3-oxo-C12-homoserine lactone (C12 HSL) induces apoptosis in *C*. *albicans*^26^ and *A*. *fumigatus*^44^. Despite this, these molecules had negligible impact on the growth of *R*. *microsporus*, confirming that other bacterial secreted factors control the growth of *R*. *microsporus*. However, high concentrations of C12 HSL (200 µM) had a marginal effect on *R*. *microsporus* growth, indicating that intra-species QS may play a role in polymicrobial biofilms.

Instead we identified that this antagonistic relationship between *P*. *aeruginosa* and *R*. *microsporus* to be the result of competition for iron. Iron acquisition is key to Mucorales pathogenesis^50^. For example, medical conditions (i.e. diabetic ketoacidosis) that result in increased serum levels of iron predispose individuals to mucormycosis^51^, whereas iron chelation therapy, or reduction in fungal iron acquisition mechanisms reduce mortality in murine models of mucormycosis^50,52^. *P*. *aeruginosa* secretes several iron binding molecules, with pyoverdine being the major siderophore with a high affinity for iron. In agreement with this, we found that exogenous pyoverdine, at concentrations equal to those secreted by *P*. *aeruginosa* in our culture conditions, was sufficient to inhibit the growth of *R*. *microsporus* to levels comparable to the bacterial supernatant. In addition, deletion of key enzymes in the biosynthesis pathways of the major *P*. *aeruginosa* siderophores was sufficient to reduce the effect of the bacterial supernatant, confirming a role for these siderophores in controlling fungal growth.

The presence of fungi has been shown to modulate the expression of siderophore biosynthetic genes in *P*. *aeruginosa*. For instance, *C*. *albicans* was described as down-regulating the production of pyoverdine and pyochelin through secreted proteins^19^. Conversely, this study has found the production of pyoverdine to be increased in response to *P*. *aeruginosa* co-cultured with *R*. *microsporus*. This is clinically important, as pyoverdine production is directly linked to virulence of *P*. *aeruginosa* and is shown to modulate the production of other toxins^53,54^.

The addition of exogenous iron or the inhibition of bacterial siderophore production only resulted in the restoration of approximately 50% fungal growth compared to media only controls, suggesting that other factors also contribute to this inhibition. However, in *Rhizopus oryzae*, iron starvation induces apoptosis^36^, suggesting that spore viability may also be affected. In agreement with this, growth of *R*. *microsporus* in 100% *P*. *aeruginosa* supernatant decreased spore viability. As such, it is possible that reduced viability may account for the inability to completely rescue fungal growth. Interestingly though, exogenous iron was able to fully restore the growth of *Mucor circinelloides*, suggesting that *M*. *circinelloides* is less susceptible to apoptosis induced by iron starvation. Differences in the ability of iron to rescue growth between Mucorales strains also suggests the presence of other potential interactions beyond iron starvation.

*R*. *microsporus* can utilise some bacterial siderophores as sources of iron within the host, such as deferoxamine (a siderophore produced by some actinomycetes) to promote its growth and virulence^55^. However, unlike deferoxamine, and potentially enterobactin, *R*. *microsporus* cannot scavenge iron from pyoverdine, which suggests that molecules with similar structure may have the potential to be used to control mucormycosis. While utilising pyoverdine itself would be problematic due to its ability to enhance *P*. *aeruginosa* virulence^54^, this siderophore could provide a starting point for the development of novel iron chelators. Given that pyoverdine has also been shown to limit the growth of other invasive fungi, such as *A*. *fumigatus*^18,56^, molecules based on pyoverdine may have wide implications for the treatment of a range of invasive fungal diseases. This is further enforced by the fact that the presence of pyoverdine in our zebrafish larval model of infection was able to reduce mortality. Similar effects have also be observed in mouse models of infection where deferasirox protects against mycormycosis^52^. Therefore, iron chelation therapy could be an important preventative treatment for mucormycosis. However, it should be noted that iron is not only important for microbial growth, but also plays essential roles in immunity^57^. Consequently, iron chelation therapy may have unexpected effects on host immunity. For example, in *C*. *elegans*, pyoverdine has been shown to induce mitochondrial damage trigging autophagy and an altered host immune response^58^. Therefore, it is important to understand the consequences these iron scavenging molecules have on the host before such therapies are applied.

Taken together, our results agree with the current understanding of Mucorales pathogenesis where iron availability is considered essential for pathogenesis. However, here we present this in a different scenario where iron availability is controlled by surrounding bacteria. Given that a high percentage of invasive mucormycosis results from burn and blast wound infections, where iron availability will be high due to tissue damage, we propose that opportunistic bacteria like *P*. *aeruginosa* will sequester iron away from the fungus restricting fungal growth. In agreement with this, burn wound exudate enhances *P*. *aeruginosa* siderophore production^59^ resulting in high concentrations of pyoverdine in the wound. However, antibiotic treatment would reduce this competition for iron, and promote fungal germination. This, coupled with natural immunosuppression following trauma could lead to aggressive secondary mucormycosis^60^. Therefore, patients that have potentially been exposed to fungal spores (i.e. soldiers with blast wounds where significant environmental contamination of the wound has occurred) should be closely monitored for secondary fungal infections. The discovery of suitable iron chelators that do not promote bacterial virulence would be advantageous in this setting to help prevent fungal infection.

## Methods

### Ethics

Zebrafish care and experiments were performed under Home Office project license P51AB7F76 and personal license I5B923969 in accordance with the Animal Scientific Procedures Act 1986.

### Strains and culture conditions

All media and chemicals were purchased from Sigma-Aldrich unless stated otherwise. For details of fungal and bacterial strains used, please see (Table 1). *R*. *microsporus* was routinely sub-cultured and maintained on Sabouraud 4% dextrose agar (SAB, Merck Millipore, Germany) and incubated for 10–14 days before use (25 °C). Bacteria were maintained on Lysogeny broth (LB) with 2% agar.

**Table 1:** Strains used in this study.

Strain	Characteristics	Source
*Rhizopus microsporus* 12.6652333	Clinical isolate	Queen Elizabeth Hospital Birmingham
*R*. *microsporus var*. *microsporus* CBS 699.68	Clinical isolate	Westerdijk Fungal Biodiversity Institute
*R*. *microsporus var*. *chinensis* CBS 631.82	Clinical isolate	Westerdijk Fungal Biodiversity Institute
*R*. *delemar* RA 99–880	Clinical isolate	Fungal Genetics Stock Centre
*Mucor circinelloides* NRRL3631	Clinical isolate	ARS Culture Collection (NRRL)
*Burkholderia cenocepecia* K56–2	Clinical isolate from cystic fibrosis	Amy Dumigan, Queen’s University Belfast
*Staphylococcus aureus* MRSA	Wild type	Anne-Marie Krachler, University of Texas
*Escherichia coli* MG1655	Wild type	Anne-Marie Krachler, University of Texas
*Pseudomonas aeruginosa* PAO1 ATCC15692	Wild type	ATCC
*P*. *aeruginosa* PAO1	Wild type	^62, 63^
*P*. *aeruginosa* ΔpchEF	PAO1, deleted pyochelin	^63^
*P*. *aeruginosa* ΔpvdD	PAO1, deleted pyoverdine	^63^
*P*. *aeruginosa* ΔpchEFΔpvdD	PAO1, deleted pyochelin and pyoverdine	^63^

### Live co-cultures

LB broth was inoculated with *P*. *aeruginosa*, *B*. *cenocepecia*, *E*. *coli*, or *S*. *aureus* and incubated for 24 h (37 °C, 200 rpm). Bacteria were washed three times with phosphate buffered solution (PBS). *R*. *microsporus* sporangiospores were harvested through flooding with PBS, washed once, and counted via haemocytometer. Spores (1 × 10^4^ spores/ml) were added to 50% SAB, 50% LB in a 96-well plate. Bacteria were added to each well at a multiplicity of infection (MOI) ratio of 1:1, 1:10, 1:50, and 1:100 and incubated for 24 h (static, 37 °C). Wells were imaged using an inverted Zeiss AxioObserver microscope (20x magnification) and the number of germinated spores per field of view quantified. Germination was defined as the point in which the germ tube reached the same size as the spore diameter.

### Spore germination when exposed to bacterial supernatants

Bacterial cultures were prepared as previously detailed and grown to at least stationary phase (OD_600_ > 3.0). Cultures were centrifuged (3220 × g, 10 min) and the resulting supernatant filter sterilised. Sterile supernatants were stored at −80 °C until required. Spores (1 × 10^4^/ml) were added to a 96-well plate containing either 50% SAB and 50% LB broth, or 50% SAB and 50% supernatant. Fungal growth was determined by endpoint analysis using OD_600_ as a quantifier of growth (FLUOstar Omega plate reader).

To investigate the role of iron restriction, ferric chloride (100 mM) was diluted to 1, 10, 50, 100, 200, and 500 µM in supernatants. The iron was allowed to associate with any chelating molecules for 15 min before the addition of an equal volume of SAB. Wells containing the SAB/supernatant mixture without iron were included as controls.

### Live cell imaging

Live-cell imaging was performed for 12–18 h at 37 °C with humidity using a Zeiss AxioObserver microscope (20x magnification). Images were taken every 10 min to create a time-lapse movie, and the percentage of germinated spores in each field of view was determined.

### Exposure of pre-germinated spores to *P*. *aeruginosa* supernatant

Spores were harvested and added to 500 µl of SAB broth at a concentration of 1 × 10^6^ spores/ml in triplicate in a 24 well plate. Spores were incubated statically for 4–5 h at 37 °C until germlings emerged and then either 500 µl of *P*. *aeruginosa* supernatant or 500 µl LB was added. The plate was incubated for 18 h at 37 °C, and the endpoint absorbance (OD_600_) of each well measured.

### Viability of spores exposed to *P*. *aeruginosa* supernatant

*R*. *microsporus* spores (1 × 10^6^ spores/ml) were exposed to 100% *P*. *aeruginosa* supernatant for 96 h (statically, 37 °C). Every 24 h, 100 spores were plated on SAB agar and incubated at 25 °C for 24 h. Following incubation, the number of viable spores were counted and compared to 0 h control plates.

### Pyocyanin secretion

*P*. *aeruginosa* supernatants were prepared as described previously. Absorbance (690 nm) was measured using a FLUOstar Omega plate reader and compared to a pyocyanin standard curve.

### RNA extraction of fungi

*R*. *microsporus* spores (2.5 × 10^6^ spores/ml) were exposed to SAB/LB (50:50) (media only control) or 50% PAO1 supernatant. Flasks were incubated statically at 37 °C for 7 h. Spores were centrifuged (1,811 × g, 3 min), snap frozen in liquid nitrogen, and stored at −80 °C. When ready to extract RNA, 1 ml of TRIzol (Invitrogen) was added to each sample and thawed on ice. These samples were homogenised as before. Chloroform (200 µl) was added to each sample, vortexed thoroughly, and centrifuged at 4 °C, 9,400 × g for 15 min. The aqueous layer was collected, and an equal volume of 100% ethanol was added. 700 µl of this was transferred to RNeasy columns, and the RNeasy Mini Plus Kit (Qiagen) protocol followed according to manufacturer guidelines. The RNA concentration and quality were measured using a spectrophotometer.

### RNA extraction of bacteria

*P*. *aeruginosa* (1 × 10^8^ CFUs/ml) were exposed to 50:50 SAB/LB (+/−100 μM FeCl_3_) or *R*. *microsporus* spores (1 × 10^6^ spores/ml, +/−100 μM FeCl_3_). Flasks were incubated at 37 °C and 50 rpm for 7 h. Cultures were centrifuged (6,000 × g for 3 min), snap frozen in liquid nitrogen, and stored at −80 °C. The RNeasy Mini Plus Kit (Qiagen) protocol for purification of total RNA from bacteria was followed according to manufacturer guidelines. The RNA concentration and quality were measured using a spectrophotometer.

### Quantitative Reverse Transcriptase PCR

qRT-PCR was performed using an iTaq Universal SYBR Green One-Step Kit (Bio Rad) using 50 ng RNA with a total reaction volume of 20 μl. Protocol was followed according to manufacturer’s recommendations. *FTR1* was amplified using the forward primer (5′-GTGGTGTCTCCTTGGGTGTT-3′) and reverse primer (5′-CCACCACGGTAGATGAGGA-3′). This was normalised to 18 s rRNA using the forward primer (5′-GGCGACGGTCCACTCGATTT-3′) and reverse primer (5′-TCACTACCTCCCCGTGTCGG-3′).

*PvdS* was amplified using the forward primer (5′-ACCGTACGATCCTGGTGAAG-3′) and reverse primer (5′- TGAACGACGAAGTGATCTGC-3′). *PchA* was amplified using the forward primer (5′- CTGCCTGTACTGGGAACAGC-3′) and reverse primer (5′-GCAGAGCAATTGCCAGTTTT-3′). These were normalised to *rpoD* using the forward primer (5′-GGGCGAAGAAGGAAATGGTC-3′) and the reverse primer (5′-CAGGTGGCGTAGGTGGAGAA-3′).

### Quantification of overall siderophore production

Siderophore concentrations in bacterial supernatants were quantified by using the SideroTec Assay Kit (Emergen Bio) according to the manufacturer recommendations.

### Zebrafish infections

Adult wild type (AB) *Danio rerio* zebrafish were maintained at the University of Birmingham Aquatic Facility in recirculating tanks with 14 h light/10 h dark cycles at 28 °C. Adult zebrafish naturally spawned overnight in groups of 11 fish (six female, five males). Embryos were transferred to E3 medium (5 mM NaCl, 0.17 mM KCl, 0.33 mM CaCl_2_, 0.33 mM MgSO_4_, pH 7) with 0.3 µg ml^−1^ methylene blue and 0.003% 1-phenyl-2-thiourea (PTU) for the first 24 hours post fertilisation (hpf) and maintained at 32 °C.

Hindbrain injections were performed as previously described^61^. Sample sizes were calculated via power analysis using an alpha value of 0.05, power of 80%, mean effect size of 4.2%, and standard deviation of 8%, based on preliminary data and standards accepted by the zebrafish infection community. At 24 hpf larvae were manually dechorionated and anaesthetised (160 µg ml^−1^ Ethyl 3-aminobenzoate methanesulfonate salt [Tricaine]). *R*. *microsporus* spores were suspended in either polyvinylpyrrolidone (PVP, 10% in PBS + 0.05% phenol red), PVP + 80 µg ml^−1^ pyoverdine, dimethyl sulfoxide (DMSO, solvent for enterobactin) or 33.5 μg ml^−1^ enterobactin at a concentration of 5 × 10^6^ spores/ml. Suspended spores (2 nl) were injected into the hindbrain via the otic vesicle to achieve a dose of 50 spores/larva. Control larvae were injected with either PVP only or PVP + 80 µg ml^−1^ pyoverdine. Any fish that did not survive the injection process were removed. Survival was recorded every 24 h until larvae were sacrificed at 5 dpf (96 hours post infection) through 10x overdose of Tricaine. For the pyoverdine experiment, data were pooled from four separate experiments with a total of 87, 80, 80, and 81 fish for control, pyoverdine only, spores only, and spores + pyoverdine, respectively. For the enterobactin experiment, data were pooled from two separate experiments with a total of 42, 44, 46, 43, and 50 fish for control, DMSO, enterobactin only, spores only, and spores + enterobactin, respectively.

### Statistical analysis

Each experiment was performed with at least two technical and two biological replicates. Microsoft Excel 2016 and GraphPad Prism 6 were used to record and analyse data. Statistical tests used are indicated in figure legends. All analysis was performed on non-normalised raw data or arcsine transformed data where appropriate. A p-value of p < 0.05 was considered to indicate statistical significance. Statistical significance is indicated by *p < 0.05, **p < 0.01, and ***p < 0.001.

## Supplementary information

Video 1

Video 2

Supplemental Figures 1, 2, and 3

## Data Availability

The datasets generated during this study are available from the corresponding author upon reasonable request.

## References

[1] Alvarez E (2009). Spectrum of zygomycete species identified in clinically significant specimens in the United States. J. Clin. Microbiol..

[2] Roden MM (2005). Epidemiology and outcome of zygomycosis: A Review of 929 reported cases. Clin. Infect. Dis..

[3] Waldorf AR, Levitz SM, Diamond RD (1984). *In vivo* bronchoalveolar macrophage defense against *Rhizopus oryzae* and *Aspergillus fumigatus*. J. Infect. Dis..

[4] Ibrahim AS, Spellberg B, Walsh TJ, Kontoyiannis DP (2012). Pathogenesis of mucormycosis. Clin. Infect. Dis..

[5] Spellberg B, Edwards J, Ibrahim A (2005). Novel perspectives on mucormycosis: Pathophysiology, presentation, and management. Clinical Microbiology Reviews.

[6] Rammaert, B. *et al*. Healthcare-associated mucormycosis. *Clin*. *Infect*. *Dis*. **54** (2012). 10.1093/cid/cir867 22247444

[7] Torres-Narbona M (2007). Impact of zygomycosis on microbiology workload: A survey study in Spain. J. Clin. Microbiol..

[8] Warkentien TE (2015). Impact of Mucorales and other invasive molds on clinical outcomes of polymicrobial traumatic wound infections. J. Clin. Microbiol..

[9] Akers, K. S. *et al*. Biofilms and persistent wound infections in United States military trauma patients: A case-control analysis. *BMC Infect*. *Dis*. **14** (2014). 10.1186/1471-2334-14-190 PMC4234323 24712544

[10] Gjødsbøl, K. *et al*. Multiple bacterial species reside in chronic wounds: A longitudinal study. *Int*. *Wound J*. **3** (2006). 10.1111/j.1742-481X.2006.00159.x PMC7951738 16984578

[11] Kalan L (2016). Redefining the chronic-wound microbiome: Fungal communities are prevalent, dynamic, and associated with delayed healing. MBio.

[12] Struck MF, Gille J (2013). Fungal infections in burns: A comprehensive review. Ann. Burns Fire Disasters.

[13] Baker R (1957). Mucormycosis; a new disease?. J Am Med Assoc.

[14] Nash G (1971). Fungal burn wound infection. J. Am. Med. Assoc..

[15] Jabra-Rizk MA, Meiller TF, James CE, Shirtliff ME (2006). Effect of farnesol on *Staphylococcus aureus* biofilm formation and antimicrobial susceptibility. Antimicrob Agents Chemother.

[16] Peleg AY (2008). Prokaryote-eukaryote interactions identified by using *Caenorhabditis elegans*. Proc. Natl. Acad. Sci. USA.

[17] Boon C (2008). A novel DSF-like signal from *Burkholderia cenocepacia* interferes with *Candida albicans* morphological transition. ISME J.

[18] Penner JC (2016). Pf4 bacteriophage produced by *Pseudomonas aeruginosa* inhibits *Aspergillus fumigatus* metabolism via iron sequestration. Microbiol. (United Kingdom).

[19] Lopez-Medina, E. *et al*. Candida albicans inhibits *Pseudomonas aeruginosa* virulence through suppression of pyochelin and pyoverdine biosynthesis. *PLoS Pathog*. **11** (2015). 10.1371/journal.ppat.1005129 PMC4552174 26313907

[20] Hogan DA (2002). *Pseudomonas-Candida* Interactions: An ecological role for virulence factors. Science (80-.)..

[21] Buffo J, Herman MA, Soll DR (1984). A characterization of pH-regulated dimorphism in *Candida albicans*. Mycopathologia.

[22] Singh P, Paul S, Shivaprakash MR, Chakrabarti A, Ghosh AK (2016). Stress response in medically important Mucorales. Mycoses.

[23] Waters CM, Bassler BL (2005). QUORUM SENSING: Cell-to-cell communication in bacteria. Annu. Rev. Cell Dev. Biol..

[24] Enjalbert B, Whiteway M (2005). Release from quorum-sensing molecules triggers hyphal formation during *Candida albicans* resumption of growth. Eukaryot. Cell.

[25] Davies DG (1998). The Involvement of cell-to-cell Signals in the development of a bacterial biofilm. Science (80-.)..

[26] Hogan DA, Vik Å, Kolter R (2004). A *Pseudomonas aeruginosa* quorum-sensing molecule influences *Candida albicans* morphology. Mol. Microbiol..

[27] Cruz MR, Graham CE, Gagliano BC, Lorenz MC, Garsin DA (2013). *Enterococcus faecalis* inhibits hyphal morphogenesis and virulence of *Candida albicans*. Infect. Immun..

[28] Allen L (2005). Pyocyanin production by *Pseudomonas aeruginosa* induces neutrophil apoptosis and impairs neutrophil-mediated host defenses *in vivo*. J. Immunol..

[29] Prince LR (2013). Subversion of a lysosomal pathway regulating neutrophil apoptosis by a major bacterial toxin, pyocyanin. J Immunol Ref. J. Immunol. Osaka Univ. Libr..

[30] Lau GW, Hassett DJ, Ran H, Kong F (2004). The role of pyocyanin in *Pseudomonas aeruginosa* infection. Trends in Molecular Medicine.

[31] Weinberg ED (1975). Nutritional immunity: Host’s attempt to withhold iron from microbial invaders. JAMA J. Am. Med. Assoc..

[32] Corbin BD (2008). Metal chelation and inhibition of bacterial growth in tissue abscesses. Science (80)..

[33] Foster J (1939). The heavy metal nutrition of fungi. Bot Rev.

[34] Ballou ER, Wilson D (2016). The roles of zinc and copper sensing in fungal pathogenesis. Curr. Opin. Microbiol..

[35] Ibrahim AS, Spellberg B, Edwards J (2008). Iron acquisition: a novel perspective on mucormycosis pathogenesis and treatment. Curr. Opin. Infect. Dis..

[36] Shirazi F, Kontoyiannis DP, Ibrahim AS (2015). Iron starvation induces apoptosis in *Rhizopus oryzae in vitro*. Virulence.

[37] Braud A, Hannauer M, Mislin GLA, Schalk IJ (2009). The *Pseudomonas aeruginosa* pyochelin-iron uptake pathway and its metal specificity. J. Bacteriol..

[38] Braud A, Hoegy F, Jezequel K, Lebeau T, Schalk IJ (2009). New insights into the metal specificity of the *Pseudomonas aeruginosa* pyoverdine-iron uptake pathway. Environ. Microbiol..

[39] Courcol RJ, Lambert Pa, Fournier P, Martin GR, Brown MR (1991). Effects of iron depletion and sub-inhibitory concentrations of antibodies on siderophore production by *Staphylococcus aureus*. J. Antimicrob. Chemother..

[40] O’Brien IG, Cox GB, Gibson F (1970). Biologically active compounds containing 2,3-dihydroxybenzoic acid and serine formed by *Escherichia coli*. Biochim. Biophys. Acta.

[41] Darling P, Chan M, Cox AD, Sokol PA (1998). Siderophore production by cystic fibrosis isolates of *Burkholderia cepacia*. Infect. Immun..

[42] Carrano CJ, Raymond KN (1979). Kinetics and mechanism of iron removal from transferrin by enterobactin and synthetic tricatechols. J. Am. Chem. Soc..

[43] Sun QN, Fothergill AW, McCarthy DI, Rinaldi MG, Graybill JR (2002). *In vitro* activities of posaconazole, itraconazole, voriconazole, amphotericin B, and fluconazole against 37 clinical isolates of zygomycetes. Antimicrob. Agents Chemother..

[44] Mowat E (2010). *Pseudomonas aeruginosa* and their small diffusible extracellular molecules inhibit *Aspergillus fumigatus* biofilm formation. FEMS Microbiology Letters.

[45] Wallace RL, Hirkala DL, Nelson LM (2018). Efficacy of *Pseudomonas fluorescens* for control of *Mucor* rot of apple during commercial storage and potential modes of action. Can J Microbiol.

[46] Hogan D (2002). a & Kolter, R. *Pseudomonas-Candida* interactions: an ecological role for virulence factors. Science.

[47] Morales, D. K. *et al*. Control of *Candida albicans* metabolism and biofilm formation by *Pseudomonas aeruginosa* phenazines. *MBio* **4** (2013). 10.1128/mBio.00526-12 PMC3560528 23362320

[48] Briard, B. *et al*. *Pseudomonas aeruginosa* manipulates redox and iron homeostasis of its microbiota partner *Aspergillus fumigatus* via phenazines. *Sci*. *Rep*. **5** (2015). 10.1038/srep08220 PMC5389140 25665925

[49] Gibson J, Sood A, Hogan DA (2009). *Pseudomonas aeruginosa-Candida albicans* interactions: Localization and fungal toxicity of a phenazine derivative. Appl. Environ. Microbiol..

[50] Ibrahim AS (2010). The high affinity iron permease is a key virulence factor required for *Rhizopus oryzae* pathogenesis. Mol. Microbiol..

[51] Artis WM, Fountain JA, Delcher HK, Jones HE (1982). A mechanism of susceptibility to mucormycosis in diabetic ketoacidosis: Transferrin and iron availability. Diabetes.

[52] Ibrahim AS (2007). The iron chelator deferasirox protects mice from mucormycosis through iron starvation. J. Clin. Invest..

[53] Meyer JM, Neely A, Stintzi A, Georges C, Holder IA (1996). Pyoverdin is essential for virulence of *Pseudomonas aeruginosa*. Infect. Immun..

[54] Lamont IL, Beare PA, Ochsner U, Vasil AI, Vasil ML (2002). Siderophore-mediated signaling regulates virulence factor production in *Pseudomonas aeruginosa*. Proc. Natl. Acad. Sci. USA.

[55] Boelaert JR (1993). Mucormycosis during deferoxamine therapy is a siderophore-mediated infection: *In vitro* and *in vivo* animal studies. J. Clin. Invest..

[56] Sass, G. *et al*. Studies of *Pseudomonas aeruginosa* mutants indicate pyoverdine as the central factor in inhibition of *Aspergillus fumigatus* biofilm. *J*. *Bacteriol*. **200** (2018). 10.1128/JB.00345-17 PMC5717155 29038255

[57] Hood MI, Skaar EP (2012). Nutritional immunity: transition metals at the pathogen-host interface. Nature Rev. Microbiol..

[58] Kang, D., Kirienko, D. R., Webster, P., Fisher, A. L. & Kirienko, N. V. Pyoverdine, a siderophore from *Pseudomonas aeruginosa*, translocates into *C*. *elegans*, removes iron, and activates a distinct host response. *Virulence*, 10.1080/21505594.2018.1449508 (2018). 10.1080/21505594.2018.1449508 PMC5955448 29532717

[59] Gonzalez MR (2016). Effect of human burn wound exudate on *Pseudomonas aeruginosa* virulence. mSphere.

[60] Kimura F, Shimizu H, Yoshidome H, Ohtsuka M, Miyazaki M (2010). Immunosuppression following surgical and traumatic injury. Surgery Today.

[61] Voelz K, Gratacap RL, Wheeler RT (2015). A zebrafish larval model reveals early tissue-specific innate immune responses to *Mucor circinelloides*. Dis. Model. Mech..

[62] Holloway BW (1955). Genetic recombination in *Pseudomonas aeruginosa*. J Gen Microbiol.

[63] Ghysels B (2004). FpvB, an alternative type I ferripyoverdine receptor of *Pseudomonas aeruginosa*. Microbiology.

