# Supplementary material for: Pseudomonas aeruginosa inhibits Rhizopus microsporus germination through sequestration of free environmental iron
Source: Sci Rep. 2019 Apr 5;9:5714. doi: 10.1038/s41598-019-42175-0 (PMC6450908; doi:10.1038/s41598-019-42175-0)
Supplement: Supplementary file 3 — Supplemental Figures 1, 2, and 3 [file 41598_2019_42175_MOESM3_ESM.pdf]

Pseudomonas aeruginosa inhibits *Rhizopus microsporus* germination through sequestration of free environmental iron

Courtney Kousser[1], Callum Clark[1], Sarah Sherrington[1], Kerstin Voelz[1*] and Rebecca A. Hall[1*]

[1]Institute of Microbiology and Infection, School of Biosciences, University of Birmingham, Birmingham, B15 2TT, UK

[*] Address correspondence to Rebecca A. Hall, r.a.hall@bham.ac.uk or Kerstin Voelz, K.Voelz@bham.ac.uk

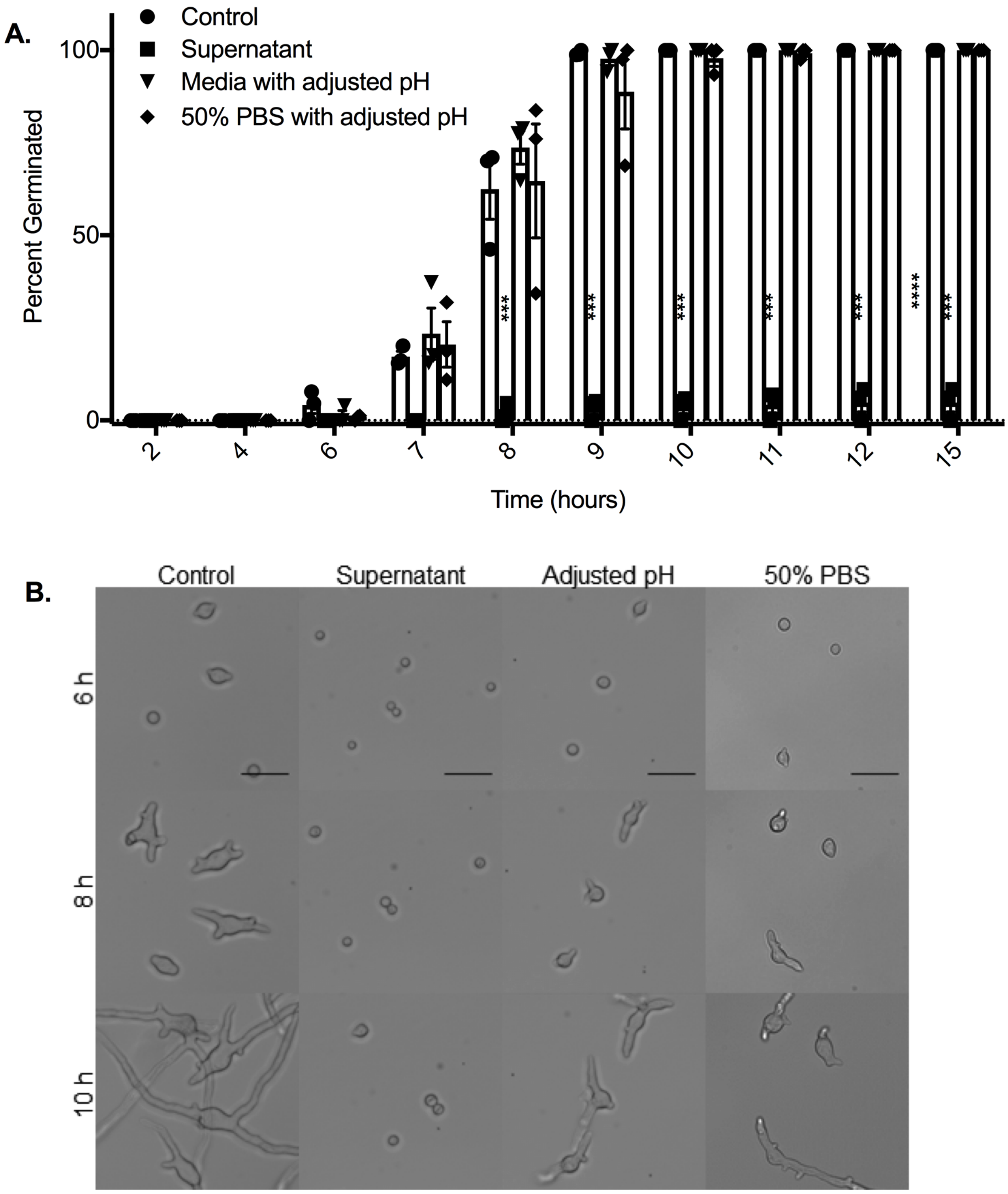
A.
Control
Supernatant
Media with adjusted pH
50% PBS with adjusted pH
Percent Germinated
100
50
0
2
4
6
7
8
9
10
11
12
15
Time (hours)
***
****
B.
Control
Supernatant
Adjusted pH
50% PBS
6 h
8 h
10 h

Supplementary Fig. S1. *Pseudomonas aeruginosa* inhibits the germination of *R. microsporus* independent of pH and macro-nutrient availability. The pH of media containing 50% PAO1 supernatant is approximately 7.3. To determine whether the inhibitory effect is caused by this change, *R. microsporus* spores were exposed to 50/50 SAB/LB with pH adjusted to 7.3. A 50% PBS control was also used to determine effects of nutrient deprivation. These conditions were imaged over time and (A) the percent of *R. microsporus* spores germinated over time was quantified and (B) representative images were obtained (n=3, Two-way ANOVA performed on arcsine transformed data). Scale bars = 50 μm. Error bars depict SEM.

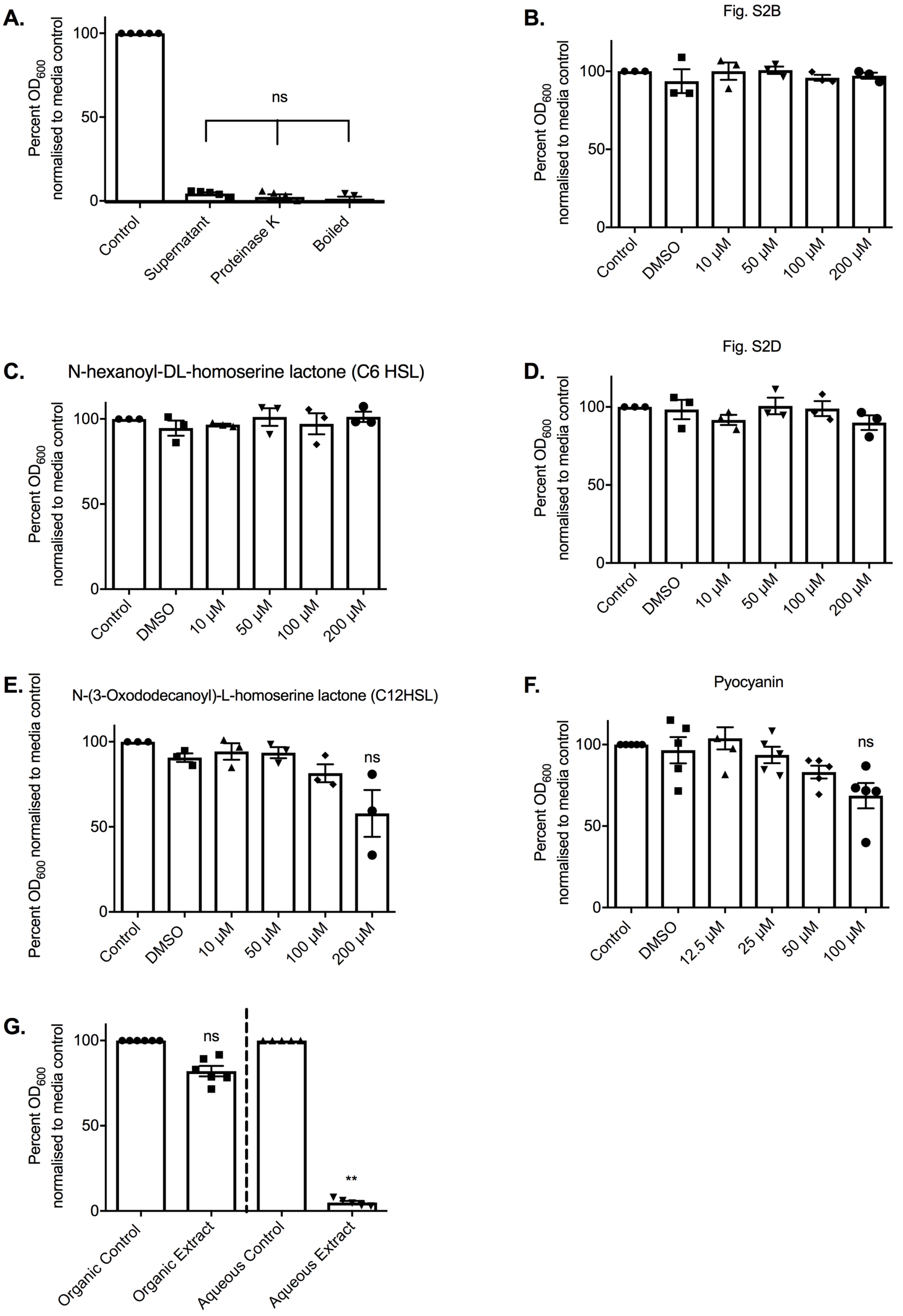
A.
Percent OD600 normalised to media control
ns
Control
Supernatant
Proteinase K
Boiled
B.
Fig. S2B
Control
DMSO
10 µM
50 µM
100 µM
200 µM
C.
N-hexanoyl-DL-homoserine lactone (C6 HSL)
D.
Fig. S2D
E.
N-(3-Oxododecanoyl)-L-homoserine lactone (C12HSL)
F.
Pyocyanin
12.5 µM
25 µM
G.
Organic Control
Organic Extract
Aqueous Control
Aqueous Extract
**

Supplementary Fig. S2. Inhibition of *R. microsporus* germination is not mediated by quorum sensing molecules or pyocyanin and is a hydrophilic heat stable molecule. (A) PAO1 supernatant was treated with 150 µg ml^{-1} Proteinase K or boiled (100˚C for 1 hour) to determine whether the inhibitory factor is a heat stable protein. Spores were exposed to treated supernatants and fungal growth was quantified through absorbance (OD_{600}) after 24 h incubation at 37˚C (n=6). *R. microsporus* spores were incubated in SAB media for 24 h at 37˚C with increasing concentrations of (B) N-butanoyl-l-homoserine lactone (C4 HSL), (C) N-hexanoyl-DL-homoserine lactone (C6 HSL), (D) N-octanoyl-L-homoserine lactone (C8 HSL), (E) N-(3-oxododecanoyl)-L-homoserine lactone (C12 HSL), and (F) pyocyanin. Fungal growth was quantified by absorbance (OD_{600}) and normalised to media control (n=3). (G) Chloroform extractions were performed to separate aqueous and organic molecules. *R. microsporus* spores were exposed to the aqueous and organic extracts for 24 h and fungal growth was determined through absorbance (OD_{600}) (n=5). All data was analysed by a Kruskal-Wallis test with Dunn's multiple comparisons test. Error bars depict SEM. ** = $p < 0.01$.

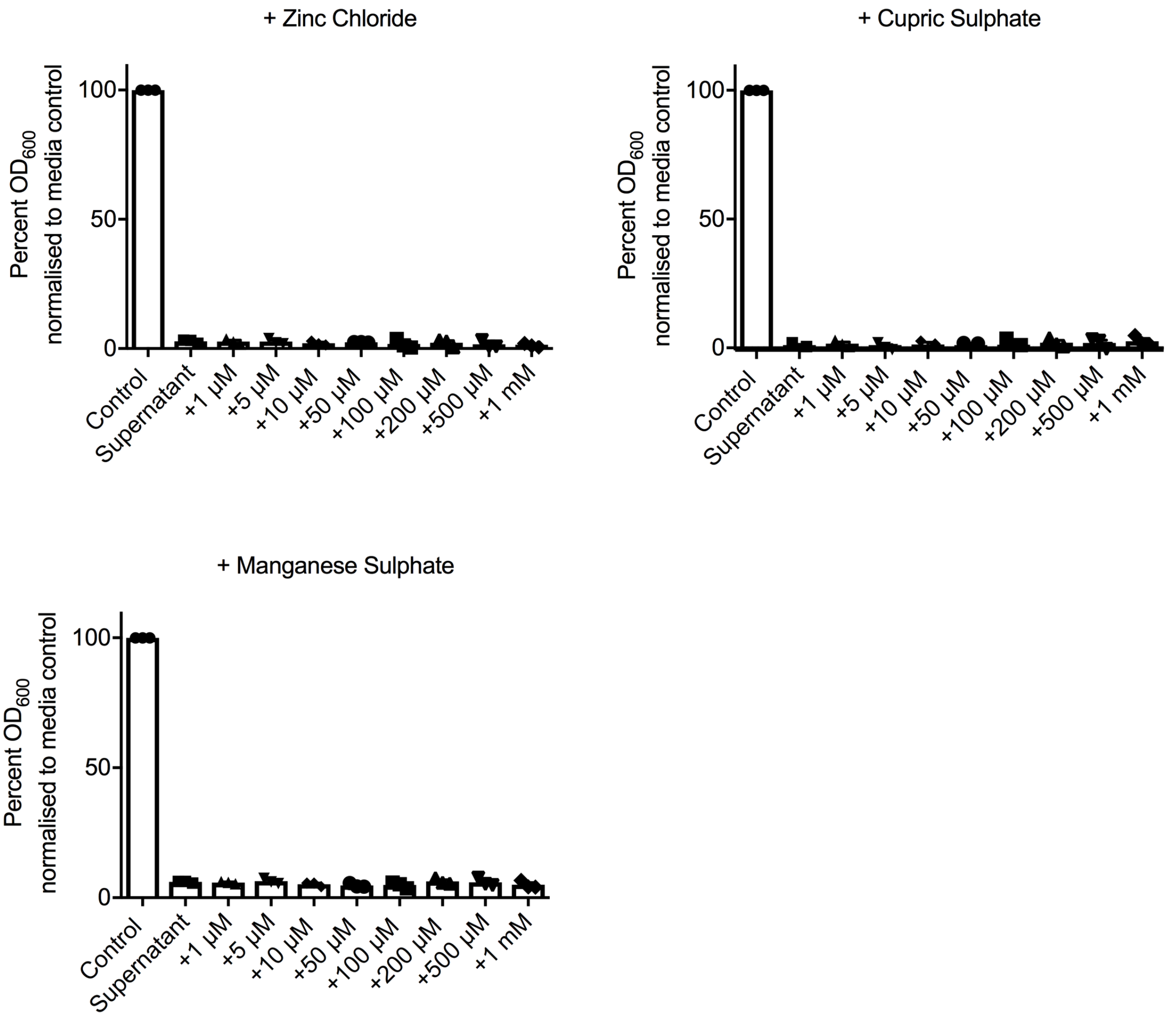

Supplementary Fig. S3. Inhibition of *R. microsporus* growth by *P. aeruginosa* supernatant is not due to zinc, copper, or manganese restriction. *R. microsporus* spores were exposed to 50% *P. aeruginosa* supernatant and spiked with increasing concentrations of zinc chloride, cupric sulphate, and manganese sulphate for 24 h statically at 37˚C. Fungal growth was measured through absorbance (OD_{600}) and normalised to media control (n=3, Kruskal-Wallis with Dunn’s multiple comparisons test).

Video 1. *R. microsporus* germination in 50% SAB, 50% LB. Images were taken every 10 min for 11 h using an inverted Zeiss AxioObserver microscope at 20x magnification. Scale bar represents 10 μM.

Video 2. The presence of *P. aeruginosa* supernatant inhibits the germination of *R. microsporus* spores. Spores were exposed to 50% *P. aeruginosa* supernatant and images were taken every 10 min for 11 h using an inverted Zeiss AxioObserver microscope at 20x magnification. Scale bar represents 10 μM.
